# HSV ICP27 hijacks host splicing factor SRSF3 to regulate pre-mRNA splicing and export for viral gene expression and immune evasion

**DOI:** 10.1371/journal.ppat.1014146

**Published:** 2026-04-10

**Authors:** Shuang Tang, Amita Patel, Kazuyo Takeda, Keith Peden, Philip R. Krause

**Affiliations:** 1 Division of Viral Products, Office of Vaccines Research and Review, Center for Biologics Evaluation and Research, Food and Drug Administration, Silver Spring, Maryland, United States of America; 2 Microscopy and Imaging Core Facility, Center for Biologics Evaluation and Research, Food and Drug Administration, Silver Spring, Maryland, United States of America; 3 Independent Consultant, Bethesda Maryland, United States of America; Tulane University School of Medicine, UNITED STATES OF AMERICA

## Abstract

HSV ICP27, a multifunctional essential immediate early (IE) viral protein, regulates both viral and host pre-mRNA processing in a gene/sequence-specific manner. Using viral mutagenesis studies, we investigated the mechanisms underlying ICP27-mediated co-transcriptional splicing inhibition. We report that ICP27 inhibits pre-mRNA splicing by hijacking host serine/arginine-rich splicing factor 3 (SRSF3), which binds to an exonic ICP27/SRSF3-responsive motif near the 5’ splice site of targeted transcripts, revealing that interaction with SRSF3 enhances both target specificity and efficiency of ICP27-mediated aberrant splicing, in a way independent of its RNA-binding RGG domain. Furthermore, ICP27 co-opts SRSF3 to promote nuclear-export of unspliced mRNA targets via the nuclear RNA export factor 1 (NXF1). Viruses with mutations both in the ICP27 N-terminal nuclear export signal (NES), via which ICP27 interacts with NXF1, and in the RGG RNA-binding domain, are defective in ICP27-mediated splicing inhibition, expression of ICP27-dependent genes, and viral growth, revealing that ICP27-mediated nuclear export of unspliced mRNA is indispensable for ICP27-mediated splicing inhibition and gene expression. Preventing U1 small nuclear ribonucleoprotein (U1 snRNP) binding by knockdown of U1-70K, a component of U1 snRNP that binds to the 5’ splice site, led both to splicing inhibition and to enhanced expression of ICP27-dependent genes. Together, these results suggest a spatiotemporal role for ICP27 in regulating sequence-specific pre-mRNA splicing by hijacking SRSF3, preventing spliceosome formation, and subsequently promoting nuclear export of aberrantly processed mRNAs containing restrictive elements including intact 5’ splice sites, which would otherwise be detained, spliced or degraded in the nucleus. We hypothesize that during latency, HSV likely takes advantage of the host mRNA processing machinery to restrict expression of randomly activated antigenic viral genes to achieve immune evasion when ICP27 is absent during latency. Upon reactivation, ICP27 is essential for ensuring both the quality and quantity of viral gene expression, enabling optimal viral replication.

## Author summary

HSV encodes abundant alternative splice junctions, but most splicing is silenced by ICP27 to ensure correct expression of short, intronless viral genes. The mechanism by which ICP27 regulates co-transcriptional splicing remains poorly understood. Here, we demonstrate that ICP27 hijacks host splicing factor SRSF3 to regulate alternative splicing by interfering with the binding of U1 snRNP to the 5’ splice site of the affected pre-mRNA, and to promote subsequent nuclear export of aberrantly processed (i.e., unspliced) mRNA by bridging between the major nuclear RNA export factor NXF1 and mRNA targets. Furthermore, U1 snRNP, which normally binds to the 5’ splice site of targeted mRNAs, also negatively regulates expression of ICP27-dependent genes. These results illuminate how ICP27 achieves target specificity, revealing that the outcome of ICP27-mediated aberrant mRNA processing reflects a delicate balance among its roles in the modification of spliceosome formation, intronic polyadenylation, and transport and decay of aberrantly processed mRNAs. Thus, pre-mRNAs of key HSV viral proteins cannot be correctly and efficiently processed by the host RNA-processing machinery in the absence of ICP27, which contributes to immune evasion during latency. Upon reactivation, ICP27 is required to ensure both the quality and quantity of these proteins.

## Introduction

HSV-1 and HSV-2, two closely related human herpesviruses, establish lifelong incurable latency in and reactivate from trigeminal ganglia and dorsal-root ganglia to cause orofacial and genital herpes, respectively. During latency in terminally differentiated neurons, expression of viral genes is repressed, except for the latency-associated transcript (*LAT*) and latency-associated miRNAs [1–3]. During acute infection, herpesvirus genes are expressed in a coordinated temporal cascade, which is characterized by three kinetic classes: immediate-early (IE or α), early (β), and late (γ). HSV infected-cell culture polypeptide 27 (*ICP27*), along with *ICP4*, are the only two IE genes essential for virus replication [1,4]. *ICP27* is known to be required for efficient expression of some viral DNA replication-related early genes and certain late viral genes as well as for viral growth [5,6]. Of the five HSV IE genes, only *ICP27* has clear homologs in all characterized mammalian herpesviruses [7]. ICP27 contains two distinct domains: an unstructured N-terminal domain and a globular C-terminal domain (CTD) [7,8]. The CTD of ICP27, a relatively conserved domain in herpesviruses, is required for homo-dimer formation [7,8].

ICP27 has been shown to regulate host and viral co-transcriptional alternative splicing and polyadenylation in a gene/sequence-specific manner by interfering with host RNA processing machinery. This leads to aberrantly spliced or aberrantly polyadenylated transcripts (here termed “aberrant splicing and polyadenylation”) from the perspective of the cell, contributing to virus-induced host shutoff and ensuring correctness of the functional coding sequences of viral genes [9–18]. There are still significant gaps in understanding the mechanism by which ICP27 mediates co-transcriptional aberrant splicing. Through its CTD, ICP27 interacts with RNA polymerase II, spliceosome components, including SAP145 and U1-70K, and multiple serine/arginine rich (SR) splicing factors (SRSFs), including SRSF3 and SRSF7 [19,17,20–24]. ICP27 has also been shown to interact with other SRSFs including SRSF5, SRSF9, SRSF1, and SRSF2 [17,25]. Through ICP27’s N-terminal RGG domain, which binds to flexible GC-rich RNAs, ICP27 directly interacts with SR protein kinase 1 (SRPK1) [17,26]. It has been hypothesized that ICP27 mediates splicing inhibition through interaction with SRPK1 by altering SR protein phosphorylation, since differential dephosphorylation of SR proteins plays a critical role in pre-mRNA splicing [17,27–30]. However, ICP27-mediated aberrant pre-mRNA splicing does not appear to be fully dependent on the RGG RNA/SRPK-1 binding domain [9,12,18], suggesting the presence of unknown mechanisms. It has been hypothesized that ICP27-mediated aberrant splicing likely occurs through an adaptor protein that enables sequence-specific interaction with mRNAs [9].

ICP27 has also been shown to play a critical role in viral mRNA export, especially of intronless/unspliced viral transcripts, many of which are essential for viral replication [31]. Through N-terminal sequences near the RGG RNA-binding domain, ICP27 directly interacts with Aly/REF, a key component of the TREX (Transcription-Export) complex that is deposited at EJCs (exon-exon junctions) and promotes export of spliced mRNAs [32–34]. However, interaction of ICP27 with Aly/REF is largely dispensable for export of most viral mRNAs, which are unspliced [35]. Via its C-terminal domain and the N-terminal leucine-rich nuclear-export signal (NES) sequences, ICP27 directly interacts with the major nuclear-export factor NXF1, and interaction with NXF1 is essential for ICP27-mediated export of RNA during HSV-1 infection [35,36].

SRSF3, also known as SRp20, is the smallest member of the highly conserved SR-rich splicing factor family that regulates alternative splicing through binding to unique exonic splicing enhancer (ESE) sequences, affects alternative 3′-polyadenylation, couples alternative splicing and polyadenylation to NXF1-mediated mRNA export [37–39], and promotes degradation of intronless mRNAs via its connection to the nuclear RNA exosomes [40,41]. SRSF3 plays important roles in tumorigenesis, cellular proliferation, cell cycle, and differentiation [42]*.* SRSF3 and SRSF7 (9G8) have been shown to contribute to efficient nuclear export of HSV transcripts [19]. ICP27 homologs EBV SM (EB2) and KSHV ORF57 have been reported to interact with SRSF3 and regulate alternative splicing [41,43].

ICP27-targeted genes have high GC content, cytosine-rich motifs, and suboptimal splice sites [9]. In this study, to better understand ICP27’s target specificity, we performed mutagenesis studies using splicing reporters and mutant viruses. We report that SRSF3 serves as a potent adaptor for ICP27, conferring target specificity and efficiency in ICP27-mediated aberrant splicing and gene expression. By hijacking SRSF3, ICP27 promotes sequence-specific aberrant splicing and nuclear export of these aberrantly processed mRNAs, thereby ensuring efficient and accurate processing and expression of these viral genes, which enables viral infection of host cells.

## Results

### ICP27 inhibits pre-mRNA splicing through interaction with SRSF3 upstream of 5’ splice site (ss)

**Fig 1:**
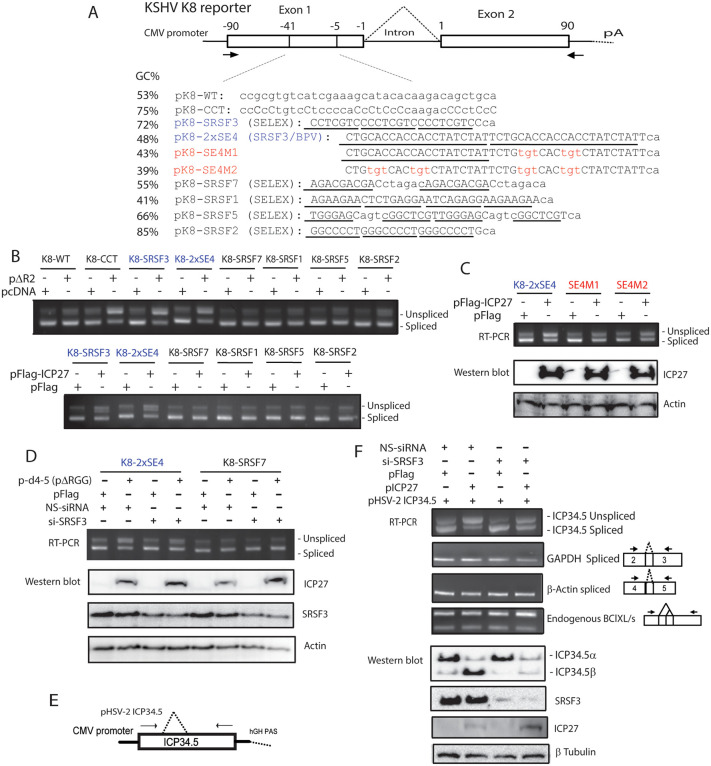
ICP27 inhibits splicing through the interaction of SRSF3 and an exonic CT- or CA-rich SRSF3-responsive motif near the 5’ splice site in a manner independent of its N-terminal RNA binding RGG domain. A) Schematic diagram of the KSHV K8 splicing reporter. Exon 1 sequences between -41 and -5 bp upstream of the 5’ ss of the K8 reporter were replaced with various SR protein-binding motifs (underlined) previously identified by SELEX. A reporter pK8-2xSE4 containing two copies of BPV exonic splicing enhancer (ESE) sequences, SE4, known to bind specifically to SRSF3 but not to other SR proteins, were also included. pK8-2XSE4 and pK8-SRSF3 are shown in blue. pK8-SE4M1 and pK8-SE4M2 (shown in red) contain point mutations in the SRSF3-binding sequence. K8-CCT is a positive control containing CT-rich mutations previously demonstrated to be sensitive to ICP27 [9]. The arrows indicate primers used for the RT-PCR in panels B-D. B) Addition of exonic CT- or CA-rich SRSF3-responsive motif in the reporter pK8-SRSF3 and pK8-2xSE4 (highlighted in blue) upstream the 5’splice site efficiently promotes ∆R2 (an HSV-2 ICP27 RNA-binding domain deletion mutant) (top panel) or full-length HSV-1 ICP27 (bottom panel)-mediated splicing inhibition, suggesting that the N-terminal RGG RNA-binding domain of ICP27 does not appear to significantly contribute to the sequence specificity of ICP27-mediated splicing inhibition of the K8 reporter. C) Mutations in the SRSF3 binding sequences in SE4M1 and SE4M2 reporters (highlighted in red) reduce ICP27-mediated splicing inhibition. D) Knocking down the expression of SRSF3 reduces ICP27-mediated splicing inhibition of K8-2xSE4 reporter. E) The pHSV-2 ICP34.5 reporter and primers used for RT-PCR used in (F). F) ICP27 inhibits splicing of the HSV-2 ICP34.5 reporter less efficiently when SRSF3 is knocked down (top of the panel). Splicing of GAPDH, β-actin and BCL XL/s are not noticeably altered by ICP27 with or without knocking down of SRSF3. Consistent with the RT-PCR results, the ratio of ICP34.5α and ICP34.5β expression is significantly altered by SRSF3 knockdown (bottom of the panel).

The RGG RNA/SRPK-1 binding domain of ICP27 is known to be dispensable for ICP27-mediated splicing inhibition. Thus, we previously hypothesized that ICP27 likely regulates pre-mRNA processing through interaction with an unknown adaptor protein that mediates its binding to the cytosine (C)-rich motif near the 5’ss to achieve target specificity, which is especially evident when the RGG RNA-binding domain, the only RNA-binding domain of ICP27, is absent [9]. Since ICP27 co-immunoprecipitates with various splicing factors including SRSF3, SRSF5 and may potentially interact with SRSF1, SRSF2 and SRSF7 (9G8) [19,17,25], we hypothesized that one or more of these splicing factors may serve as the adaptor for interaction between ICP27 and pre-mRNA. To evaluate the potential contribution of different splicing factors to ICP27-mediated splicing inhibition, we inserted multiple copies of binding motifs for different splicing factors including SRSF1 [44], SRSF2 [44], SRSF3 [45], SRSF5 [46], and SRSF7 [45], for which consensus mRNA binding motifs have been determined, into exon 1 upstream of the 5’ss of a KSHV K8 splicing reporter (Fig 1A), which has been used previously to evaluate ICP27’s role in co-transcriptional splicing [9]. ICP27 does not affect the splicing of the K8 splicing reporter (pK8-WT), and ICP27-mediated splicing inhibition depends on the sequences upstream of the 5’ss [9,18]. WT K8 reporter (pK8-WT) and a mutant K8 reporter (pK8-CCT) with C-rich sequence mutations in the 5’ exon, previously shown to enable ICP27-mediated inhibition of splicing, served as negative and positive controls [9]. The effect of a mutant HSV-2 ICP27 (∆R2) that lacks the RGG RNA-binding domain on the splicing of these K8 reporters was evaluated by co-transfection of the reporter plasmids with ICP27 plasmids, pcDNA or pFlag empty vector plasmids (as negative controls) into 293T cells. Both the HSV-2 ICP27 RNA-binding domain mutant (∆R2) and HSV-1 WT ICP27 substantially inhibit the splicing of the K8-CCT transcript but not the K8-WT transcript, which is consistent with previous findings [9]. Neither mutant HSV-2 ICP27 (∆R2) nor WT HSV-1 ICP27 substantially altered the splicing of reporters containing SRSF7, SRSF1, SRSF5 and SRSF2-binding sequences (Fig 1B, Upper Panel). However, the mutant ICP27 (∆R2) also substantially inhibited splicing of the transcripts expressed from pK8-SRSF3 but not those containing SRSF1, SRSF2, SRSF5, and SRSF7-binding motifs (Fig 1B, Upper Panel). To confirm the results, we included a K8 reporter (pK8-2xSE4) that contains two copies of bovine papillomavirus virus (BPV) exonic splicing enhancer 4 (SE4), which has much lower GC content and is known to interact with SRSF3 but not with other splicing factors or RNA-binding proteins [47,48]. Mutant ICP27 (∆R2) inhibited splicing of the K8 transcripts expressed from pK8-2xSE4 (Fig 1B, Upper Panel), suggesting that SRSF3 is a potent adaptor for ICP27 in regulating alternative splicing. Similar results were obtained when WT HSV-1 ICP27 with a Flag epitope fused to the N-terminus was transfected with the reporters (Fig 1B, Lower Panel), indicating that SRSF3 contributes to ICP27-mediated splicing inhibition regardless of whether ICP27’s RNA-binding domain that interacts with GC-rich RNA and SRPK-1 is present or not. This result also suggests that the interaction of ICP27 with GC-rich RNA or with SRPK-1 does not play a critical role in the sequence-specificity of ICP27-mediated splicing inhibition. It has been shown that addition of N-terminal fusion tags, including the Flag tag and fluorescent proteins, does not affect ICP27’s WT function [12,36]. Furthermore, mutation of the proximal CA-rich SRSF3 binding sequences in the SE4 enhancer (SE4M1 and SE4M2) significantly reduced ICP27-mediated splicing inhibition (Fig 1C). Notably, we specially designed the mutation for the SE4M reporters to be the same as previous published mutation, which was reported to specifically disrupt the interaction with SRSF3 [47]. Therefore, this study further demonstrated that the interaction of SRSF3 contributes to both the specificity and efficiency of ICP27-mediated splicing inhibition. Knockdown of SRSF3 expression by SRSF3-specific siRNA treatment significantly impaired ICP27-mediated splicing inhibition using either the K8-SE4 reporter construct (Fig 1D) or HSV-2 ICP34.5 expression plasmid (Fig 1E and F). The flag-tagged HSV-1 WT ICP27 expression plasmid (pFlag-ICP27) and an HSV-2 WT ICP27 expression plasmid (pICP27) was used in Fig 1E and 1F, respectively. In the absence of ICP27, knockdown of SRSF3 had no impact on splicing. Consistent with the RT-PCR results, when ICP27 is present, the ratio between ICP34.5β (the protein variant that is expressed from unspliced full-length ICP34.5 mRNA) and ICP34.5α (the protein variant that is expressed from spliced ICP34.5 mRNA) was significantly reduced in SRSF3-knockdown cells (Fig 1F). Indeed, the previously described ICP27-responsive motifs observed in ICP27-mediated aberrantly processed transcripts resemble the SRSF3-responsive motif identified using a SRSF3-specific siRNA and splicing array, or by iCLIP (cross-linking immunoprecipitation) studies, all sharing core cytosine-rich sequences [9,49–51]. While ICP27 N-terminal RGG RNA-binding sequences appear to play a role in splicing inhibition, they are not required, a finding consistent with previous reports that the N-terminal sequences of ICP27 are not required for its binding to SRSF3 [17]. Taken together, the data indicate that SRSF3 is required for ICP27-mediated sequence/gene specific splicing inhibition, explaining the observed mRNA sequence specificity for both HSV-1 and HSV-2 ICP27.

### The ICP27 nuclear-export signal (NES) domain, in combination with the RGG RNA binding domain, plays a role in alternative splicing

**Fig 2:**
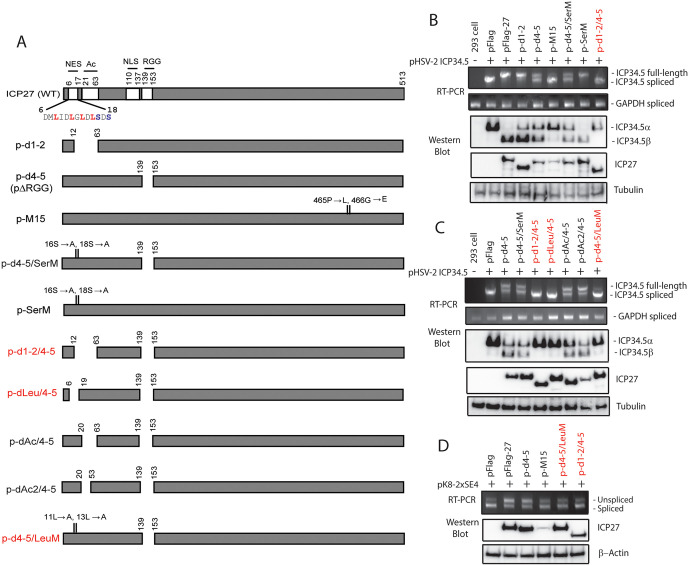
Mapping of cis-elements required for ICP27-mediated splicing inhibition using HSV-2 ICP34.5 as a reporter. The N-terminal leucine-rich NES becomes indispensable for ICP27-mediated splicing inhibition and expression of ICP34.5β encoded by unspliced mRNA when the N-terminal RGG domain is absent. A) Schematic diagrams of HSV-1 ICP27 mutant expression plasmids. B and C) RT-PCR and Western blot analysis of 293T cells co-transfected with the ICP34.5 expression plasmid and WT or ICP27-mutants. Double mutations in the N-terminal NES and RGG domains in p-d1-2/4-5, p-dLeu/4-5 and p-d4-5/LeuM (highlighted in red) abolished ICP27-mediated splicing inhibition and expression of ICP34.5β. D) Both the N-terminal RGG domain and NES are required for ICP27-mediated splicing inhibition for pK8-2xSE4. The illustrated diagrams for pHSV-2 ICP34.5 and pK8-2xSE5 reporters and primers used for RT-PCR are illustrated in Fig 1E and A, respectively. The new HSV-1 ICP27 mutant plasmids including p-d4-5/LeuM, p-dLeu/4-5 and p-d1-2/4-5 are shown in red.

Viral mutagenesis studies of ICP27 have historically yielded key insights into ICP27’s function [1,52–54]. Since deletion of the ICP27 RGG domain did not abrogate splicing inhibition, we sought to determine whether other N-terminal sequences may play a role. In previous experiments, deletion of the NES domain slightly reduced ICP27-mediated splicing inhibition [9]. The NES domain and adjacent sequences contain a leucine-rich sequence, CK2 phosphorylation sites (Ser16 and Ser18), and an acidic amino acid rich sequence (Ac) [52]. Mutations in either the leucine-rich sequence or of the CK2 phosphorylation sites resulted in nuclear retention of ICP27 [32,55]. To further understand the mechanism of ICP27-mediated splicing inhibition, we evaluated the impact of these sequences on ICP27-mediated splicing inhibition by evaluating additional HSV-1 ICP27 mutations in plasmid transfection experiments (Fig 2A). The deletion d4-5 removed the RGG RNA-binding sequences, while other deletions (including in the d4-5 background) were in the NES region. We found that mutation or deletion of the NES and its adjacent region (p-d1-2 and p-SerM) on their own had no obvious impact on the splicing of HSV-2 ICP34.5 (Fig 2B). However, dual mutation of the core NES and RGG domains (d1-2/4–5, dLeu/4–5 and d4-5/LeuM) abolished ICP27-mediated splicing inhibition of the HSV-2 ICP34.5 reporter and expression of HSV-2 ICP34.5β protein (which is expressed from unspliced mRNA), revealing a role for the core NES sequences in ICP27-mediated splicing inhibition, which was evident when the RGG RNA-binding domain was absent (Fig 2B and C). Thus, these results also suggest that the impact of the core NES on pre-mRNA splicing may be compensated for by ICP27’s functions related to the RGG domain.

Dual mutations targeting both the NES-adjacent sequences and the RGG domain (d4-5/SerM, dAc/4–5, and dAc2/4–5) had similar effects on the splicing efficiency of the ICP34.5 reporter as the RGG domain mutation alone (d4-5) (Fig 2B and C). This suggests the two phosphorylation sites (Ser16 and Ser18) and the acidic region (aa21–63), which are located slightly outside the core NES, are less critical for ICP27-mediated splicing inhibition than the core NES itself.

Compared with the full-length HSV-2 ICP34.5 expression reporter, the K8-2SE4 reporter is a simplified model for ICP27-mediated aberrant splicing (Fig 1). The K8-2SE4 reporter contains only the SE4 element with a normal GC content of 48%, which interacts with SRSF3 but not with other splicing factors [47,48]. Therefore, we also evaluated the role of the NES in the splicing of the K8-2SE4 reporter and confirmed that dual mutation in the core NES and RGG domains (d1-2/4–5 and d4-5/LeuM) also abolished ICP27-mediated splicing inhibition of the pK8-2xSE4 reporter (Fig 2D).

### Both the RGG and core NES sequences of ICP27 are required for efficient viral growth and expression of ICP27-dependent genes

**Fig 3:**
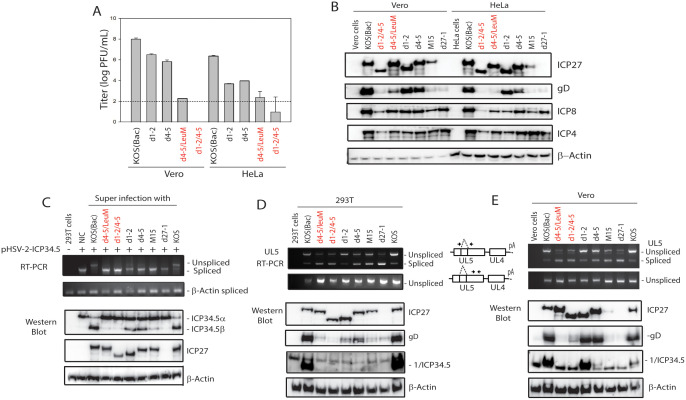
Viruses with mutations in both the RGG and core NES sequences of ICP27 are defective in viral growth, and less efficient in inhibiting of the pre-mRNA splicing and promoting gene expression of ICP27 targeted genes. A) Viruses with mutations in both the RGG and core NES sequences of ICP27 are defective in viral growth. Viral growth was measured in Vero and HeLa cells infected with WT or ICP27 mutants. Titration was performed in V27 cells in triplicates. ICP27 mutants, including d4-5/LeuM and d1-2/4-5 with the mutations illustrated in Fig 2A, were constructed using an HSV-1 KOS bacterial artificial chromosome (BAC) system. Both mutants were verified by sequencing. KOS (bac) is WT KOS after removal of the BAC cassette, and d27-1 is an ICP27 deletion mutant. The detection limit of the assay is 100 PFU/mL. B) Expression of ICP27-dependent genes (e.g., gD) in d4-5/LeuM and d1-2/4-5 infected cells are drastically reduced to a level comparable to d27-1 or m15, a known non-functional mutant with two-amino acid mutations at the CTD, which is believed to perturb formation the homodimer [8]. ICP8 and ICP4, known to be less dependent on ICP27, serve as an infection control. C) Superinfection with mutant viruses with mutations in both the RGG and core NES sequences could not inhibit splicing of the HSV-2 ICP34.5 reporter and promote expression of ICP34.5β. RT-PCR and Western blot analyses were performed in 293T cells transfected with the HSV-2 full-length ICP34.5 expression plasmid for 16 hours, followed by superinfection with WT and ICP27 mutants. Viruses with mutations in both the RGG and core NES sequences of ICP27 are less efficient in the inhibition of the splicing of UL5 pre-mRNA and protein expression of ICP27-dependent genes including ICP34.5 and gD in 293T cells (D) and Vero cells (E). Primers used in the RT-PCR that map across the alternatively spliced intron and within exon 2 of UL5 are illustrated in Panel D on the right. The two new HSV-1 ICP27 mutant viruses d4-5/LeuM and d1-2/4-5 are shown in red.

To confirm the function of NES and RGG in pre-mRNA processing, we constructed two mutant viruses (d4-5/LeuM and d1-2/4–5) carrying the same mutations as illustrated in Fig 2A. Since the NES domain might have cell-type dependent effects [56], we evaluate the viral growth in both Vero and HeLa cells. d4-5/LeuM and d1-2/4–5 are significantly attenuated, and the growth rates for both were near the detection limit in both cell lines (Fig 3A). It appears that d1-2/4–5 is more defective in viral growth (Fig 3A). Consistent with the defective viral growth, expression of gD, whose expression is dependent on ICP27, in d1-2/4–5 and d4-5/LeuM infected cells was substantially reduced to levels similar to those observed with the ICP27 deletion mutant (d27-1) and m15 (Fig 3B). This suggests that the biological function of the NES domain partially overlaps with that of the RGG domain and is largely dispensable when the RGG domain is present, which is consistent with the findings from the reporter experiments (Fig 2). When one of the two domains is mutated, the biological function of the other domain becomes essential. ICP8 and ICP4, whose expression is less dependent on ICP27, served as infection controls.

### The RGG and core NES sequences of ICP27 in HSV-1 are required for ICP27’s role in splicing inhibition in infected cells

We next evaluated the impact of the NES mutations on pre-mRNA splicing in three different cell lines, including 293T, HeLa and Vero, infected with d1-2/4–5 and d4-5/LeuM mutant viruses. In 293T cells pre-transfected with the HSV-2 ICP34.5 expression plasmid and infected with WT or ICP27 mutants, dual RGG/NES mutants did not inhibit the splicing of ICP34.5 or promote expression of ICP34.5β (Fig 3C). In both 293T and Vero cells infected with WT or ICP27 mutants, the dual RGG/NES mutants also did not inhibit the splicing of UL5 (Fig 3D and E), a recently identified spliced viral gene for which splicing is largely silenced by ICP27 [10]. Consistent with the results in Fig 3B, the dual RGG/NES mutants did not promote expression of ICP27-dependent genes including gD and ICP34.5 in either 293T or Vero cells. The impact of NES and RGG sequences nonetheless had some cell-type dependence, consistent with the observation that the NES may have a cell-type specific impact on viral gene expression [56]. For example, mutation of NES (d1-2) or RGG (d4-5) had greater impact on the expression of gD in 293T cells than in Vero or HeLa cells. Deletion of the NES domain (d1-2) substantially reduced expression of HSV-1 ICP34.5 in 293T cells but not in Vero cells (Fig 3 D and E). However, dual mutation of the NES and RGG domains (d1-2/4–5 and d4-5/LeuM) abolished the expression of both gD and ICP34.5 in all three cell lines (Fig 3B, D and E).

### Infection with the ICP27 dual NES/RGG mutants induces increased colocalization of SRSF3 with either ICP27 or NXF1 in nuclear speckles

**Fig 4:**
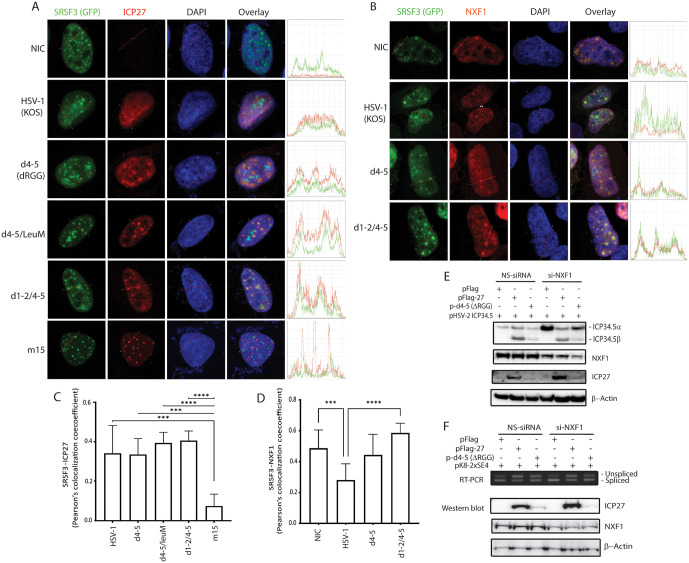
The core NES sequences of ICP27 contribute to its role in splicing inhibition, likely through promoting the export of ICP27-targeted unspliced mRNAs via interaction with NXF1, especially evident when the RNA-binding domain of ICP27 is absent. A) Mutations in both the RGG and core NES sequences of ICP27 do not affect the co-localization of ICP27 with SRSF3. HeLa cells pre-transfected with a SRSF3-GFP expression plasmid for 48 hours were infected with WT and ICP27 mutant viruses as indicated. Cells were fixed at 6 hours post infection (hpi) and incubated with anti-ICP27 antibody (H1113). B) NXF1 is co-localized with SRSF3, and infection with WT or ICP27 mutants does not appear to alter the colocalization pattern of SRSF3 with NXF1. HeLa cells were transfected with SRSF3-GFP and myc-NXF1 plasmids 48 hours prior to infection with or without WT and ICP27 mutants as indicated. Cells were fixed at 6 hpi and incubated with an anti-MYC tag antibody. White open arrow indicates membrane. Pearson’s co-localization coefficient (PCC) analyses were also performed to examine the nuclear spatial relationship between SRP20 and ICP27 shown in (C) and between SRP20 and NXF1 shown in (D). *** indicates P value <0.005 and **** indicates P value <0.0001. E-F) Reduced expression of NXF1 by siRNA impaired ICP27-mediated splicing inhibition for the HSV-2 ICP34.5 reporter (E) or the K8-2xSE4 reporter (F), especially for ICP27 ∆RGG mutant (d4-5). Western blot analysis of 293T cells co-transfected with the HSV-2 full-length ICP34.5 expression plasmid with WT and mutant ICP27 plasmids for 16 hours is shown in (F). RT-PCR and western blot analysis of 293T co-transfected with K8 2xSE4 reporters and WT and mutant ICP27 plasmids are shown in (F).

To determine whether the dual NES/RGG mutations affected the interaction between SRSF3 and ICP27, we performed an immunofluorescence study in HeLa cells. In non-infected cells, SRSF3 was localized to nuclear speckles, the structure of which was slightly modified upon HSV infection. ICP27 bearing all N-terminal ICP27 mutations including d4-5, d4-5/LeuM and d1-2/4–5, but not the C-terminal mutant m15, remained colocalized with SRSF3 in nuclear speckles of infected cells (Fig 4A). m15 contains two single-amino acid mutations at the CTD that are believed to perturb formation of the ICP27 homodimer [8]. m15 could no longer mediate splicing inhibition or promote intronic polyadenylation (Fig 2) [9]. These results are also consistent with the findings that the N-terminal domains of ICP27 are dispensable for the interaction of ICP27 with SRSF3 and that ICP27 binding to SRSF3 involves ICP27 C-terminal sequences [17]. Therefore, the mechanism by which dual mutations in the core NES and RGG domains (d1-2/4–5 and d4-5/LeuM) abolish ICP27-mediated splicing inhibition is not due to a change to the interaction between SRSF3 and ICP27. Compared with WT ICP27, viral mutants with mutations in the ICP27 RGG domains (d4-5, dLeuM/d4-5 and d1-2/4–5) appeared to be more closely colocalized with SRSF3 in nuclear speckles in infected cells (Fig 4A), suggesting that as expected, the dual mutations in the NES and RGG domain do not affect its interaction with SRSF3; however, there may be a change of mobility of these ICP27 mutants. We thus hypothesize that the change of mobility of these dual NES/RGG mutants may lead to impaired nuclear export.

SRSF3 has been shown to be a potent adaptor for the major nuclear-export factor NXF1, conferring sequence specificity to RNA binding by NXF1 [37]. Both NXF1 and SRSF3 are implicated in nuclear export of intronless transcripts [57,58]. NXF1 has been shown to play a critical role in ICP27-mediated nuclear export of viral mRNAs, and both the N-terminal NES and C-terminal domains of ICP27 are required for direct interaction with NXF1 [35,59]. Thus, we next evaluated the colocalization pattern of SRSF3 and NXF1 in the context of virus infection. We found that SRSF3 and NXF1 were colocalized in nuclear speckles (Fig 4B). Neither WT nor ICP27 mutant viruses obviously altered the colocalization profile of NXF1 and SRSF3, which is consistent with a previous observation that ICP27 does not alter the localization of NXF1 [35]. ICP27 interacts with SRSF3 via its CTD and with NXF1 with its N-terminal NES, and SRSF3 can interact with NXF1. Therefore, SRSF3 likely functions as a potent adaptor bridging ICP27, NXF1 and target mRNAs for export, thus conferring sequence specificity to RNA binding by NXF1.

Pearson’s co-localization coefficient (PCC) analyses were also performed to examine the nuclear spatial relationship between SRSF3 and ICP27 (Fig 4C) and between SRSF3 and NXF1 shown in (Fig 4D). SRSF3 and ICP27 (except for the inactive m15 mutant) displayed punctate nuclear distributions that frequently co-localized in nuclear speckles (Fig 4C). Dual mutation of the NES and the RGG domain slightly enhanced the colocalization of ICP27 with SRSF3 (Fig 4C). In non-infected control cells, overexpressed SRSF3 and NXF1 displayed punctate nuclear distributions that frequently co-localized in nuclear speckles (Fig 4B and D). Following infection with WT HSV-1, NXF1 was detected in nuclear puncta and also frequently localized at the nuclear membrane (indicated by an open arrow in Fig 4B). Consistent with this distribution, SRSF3 showed significantly reduced co-localization with NXF1 in cells infected with WT HSV-1 as compared with that in non-infected cells and in cells infected with d1-2/4–5 (Fig 4D*)*. This observation aligns with previous findings showing that overexpressed NXF1 exhibits greater localization to nuclear puncta rather than the cell membrane in cells co-transfected with C-terminal ICP27 mutants compared to wild-type ICP27 following infection with a ICP27 deletion mutant [59]. Our results demonstrated that WT HSV-1 infection redistributed NXF1 within the nucleus, and this redistribution was completely dependent on functional NES and RGG domains in ICP27, as the dual mutant (d1-2/4–5) abolished this effect. In cells infected with dual NES/RGG mutants, NXF1 showed enhanced colocalization with SRSF3 in nuclear speckles, indicating retention in splicing compartments. This observation suggests that the impaired splicing inhibition observed in dual NES/RGG mutants (d1-2/4–5 and d4-5/LeuM) likely results from their inability to recruit NXF1 to facilitate nuclear RNA export of ICP27-mediated aberrant transcripts.

### Efficient ICP27-mediated splicing inhibition is NXF1 dependent

We further investigated the impact of NXF1 on the splicing of ICP27-targeted genes in NXF1 knockdown cells using HSV-2 ICP34.5 expression vectors. When NXF1 was knocked down by siRNA, both WT ICP27 and d4-5 favored expression of the ICP34.5α protein (expressed from spliced ICP34.5 mRNA) (Fig 4E), indicating that interaction of NXF1 with ICP27 contributes to nuclear export of unspliced ICP34.5 mRNA (leading to expression of ICP34.5β). Similarly, in cells co-transfected with the pK8-2xSE4 splicing reporter (illustrated in Fig 1) and WT or RGG mutant, knockdown of NXF1 reduced ICP27-mediated splicing inhibition (Fig 4F). Since the K8-2SE4 reporter is a simplified model for ICP27-mediated aberrant splicing (Fig 1 and 2), these results indicated that prevention of spliceosome formation by ICP27 via SRSF3 alone did not ensure efficient splicing inhibition, and subsequent nuclear export of targeted unspliced mRNAs is also required for ICP27-mediated splicing inhibition. When the RGG domain is absent (d4-5), the interaction of the NES of ICP27 and NXF1 became indispensable for nuclear export of unspliced mRNA bridged by SRSF3.

### ICP27 counteracts the activity of U1 snRNP to promote expression of target genes

**Fig 5:**
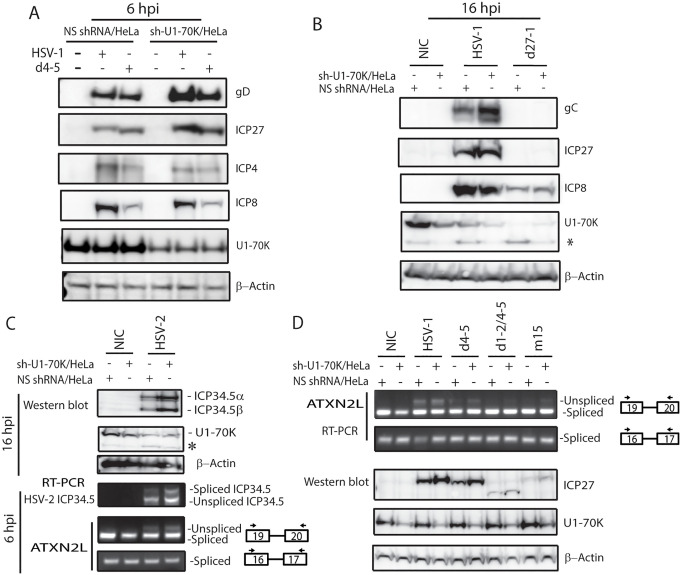
ICP27 counteracts U1 snRNP’s role in negatively regulating expression of ICP27-dependent viral genes. A) In U1-70K-knockdown HeLa cells, expression of ICP27 dependent genes including gD, as well as ICP27 itself, are significantly enhanced. In contrast, expression of genes that are independent on ICP27, such as ICP4 and ICP8 are not significantly altered. U1-70K shRNA or Non-specific shRNA HeLa stable cells were infected with or without HSV-1 or d4-5 at 5 MOI. Total proteins were prepared at 6 hpi. B) In U1-70K-knockdown HeLa cells infected with WT HSV-1, expression of HSV-1 gC, an ICP27-dependent true late gene, is significantly increased at the late viral infection (16 hpi). Expression of gC is expressed below the detectable level, and moderate reduction of ICP8 is also observed in both U1-70K down HeLa cells and control cells. *indicates a minor band specific to the U1-70K antibody likely due to degraded fragment during viral infection. C) Reduced expression of U1-70K enhances HSV-2 ICP27’s role in promoting ICP34.5 expression. U1-70K shRNA or non-specific shRNA HeLa stable cells were infected with or without HSV-2 strain 333. U1-70K is also degraded at late viral infection. *indicates a U1-70K antibody-specific minor band. D) Knockdown of U1-70K slightly enhances ICP27-mediated splicing inhibition. U1-70K shRNA or non-specific shRNA HeLa stable cells were infected with or without WT or ICP27 mutant viruses as indicated for 6 hours in the figure.

In the absence of ICP27, neither NXF1 nor SRSF3 was able to promote export of ICP27-mediated aberrantly processed mRNAs, e.g., full-length unspliced HSV-2 ICP34.5 that encodes ICP34.5β (Fig 2B and C) or full-length unspliced HSV-1 ICP3.45 and gD (Fig 3), suggesting that these aberrantly processed mRNAs may have features that cause them to be detained within the nucleus and ultimately degraded when ICP27 is absent. We previously found that ICP27 stimulates expression of hundreds of GC-rich intronic polyadenylated transcripts and all ICP27-mediated aberrantly processed mRNAs contain an intact 5’ splice site, which interacts with U1 snRNP [9]. Additionally, HSV encodes abundant hidden splice junctions and cryptic 5’ splice sites [10]. Interaction of U1 snRNP to the 5’ splice sites is not only required to initiate splicing but also to prevent pre-mRNAs from premature cleavage and polyadenylation, and plays a role in quality control of the aberrantly processed mRNAs that containing 5’splice sites [60–62]. Intact 5’ splice sites have been shown to be a nuclear RNA degradation code for host intronic polyadenylated transcripts to be detained in nuclear speckles and degraded within the nucleus [61,63]. To evaluate the impact of the presence of intact 5’ splice sites in the ICP27-targeted transcripts, we next established a stable U1-70K stable knockdown HeLa cell line to evaluate the role of U1-70K, the key component of U1 snRNP, in ICP27-mediated aberrant pre-mRNA processing. We found that expression of viral proteins such as ICP8 and ICP4, for which expression is less dependent on ICP27 [5], was not impacted by knockdown of U1-70K (Fig 5A and B). However, expression of gD, an ICP27-dependent viral gene (Fig 3), was upregulated in both WT and d4-5 mutant - infected U1-70K-knockdown cells (Fig 5A). Expression of gC, a true late-viral gene for which expression is strictly dependent on ICP27, was also significantly enhanced in U1-70K knockdown cells at late viral infection (Fig 5B). Similarly, in U1-70K-knockdown cells, expression of both HSV-2 ICP34.5α and β (derived from spliced and unspliced ICP34.5 mRNAs, respectively) increased, which is consistent with increased expression of both spliced and unspliced mRNAs (Fig 5C). Significant degradation of U1-70K during late viral infection in both WT- and the ICP27 mutant-infected cells (Fig 5B and C) suggested that U1-70K is likely a critical target for HSV infection. It appears that HSV employs different mechanisms at early infection (via ICP27) and at late infection (via an unknown pathway) to inhibit U1-70K activity, contributing to host shut-off and expression of viral genes. Knockdown of U1-70K only slightly enhanced ICP27-mediated aberrant viral (HSV-2 ICP34.5) or cellular splicing (Fig 5C and D), likely because the reduced level of U1-70K remained adequate to permit splicing to occur in the knockdown cells. Indeed, U1-70K was essential for alternative splicing, and there were no changes in viral-growth rate or basal splicing in the U1-70K-knockdown cells as compared with the control cells. However, ICP27-dependent viral genes like ICP34.5α and β were expressed at higher levels in infected U1-70K-knockdown cells, implying that in addition to U1-70K’s direct role in regulating splicing, U1-70K has an additional role in limiting expression of aberrantly processed mRNAs, and counteracting this activity of U1-70K plays an important role in expression of ICP27-dependent genes. These results also suggest that when ICP27 is absent during latency, ICP27-dependent genes are subjected to U1 snRNP-mediated nuclear RNA surveillance machinery, contributing to viral immune evasion. During reactivation, ICP27 counteracts U1 snRNP’s function in pre-mRNA splicing inhibition and nuclear RNA surveillance, promoting aberrant splicing and efficient expression of ICP27-dependent genes.

### Proposed spatiotemporal model for ICP27-mediated co-transcriptional aberrant pre-mRNA processing

**Fig 6:**
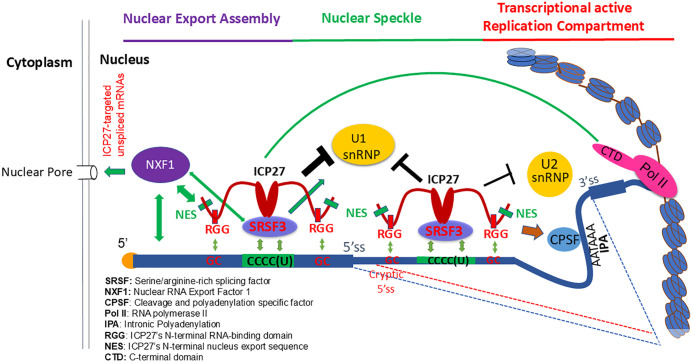
Proposed spatiotemporal roles for ICP27-mediated sequence-specific co-transcriptional aberrant pre-mRNA processing. Through interaction with the CTD of RNA polymerase II, ICP27 and SRSF3 are able to associate with nascent pre-mRNAs [23,24,64]. ICP27 regulates pre-mRNA splicing through both direct binding (through its RGG domain) and indirect binding via SRSF3 (through its CTD) to the ICP27/SRSF3-responsive motif, which is located upstream of the 5’ splice site in targeted transcripts, preventing the spliceosome formation initiated by binding of U1 snRNP and U2 snRNP to the splice sites of the impacted intron. ICP27-mediated aberrant splicing is also dependent on ICP27’s role in promoting the transport of unspliced mRNA from nuclear speckles to the cytosol via NXF1 (through the NES of ICP27), SRSF3 and direct or indirect interaction with targeted mRNA. Mutation of both the NES and RGG domain completely abolished ICP27’ role in redistribution of NXF1 in the nucleus. An extended stay of ICP27-targeted unspliced pre-RNAs in nuclear speckles likely leads to their eventual splicing, or degradation by the nuclear mRNA surveillance machinery including those mediated by U1 snRNP through binding to the intact 5’ splice site. ICP27 is required to counteract U1 snRNP-mediated mRNA surveillance activities for efficient expression of ICP27-dependent viral genes. Additionally, direct or indirect binding via SRSF3 to the intronic ICP27/SRSF3-responsive motif may also contribute to splicing inhibition and intronic polyadenylation by preventing U1 and U2 snRNPs from binding to the 5’ and 3’ splicing sites, blocking SRSF3’s role in inhibition of proximal PAS usages and affecting the interaction of ICP27 with the polyadenylation factors (CPSF).

Together, our results revealed a spatiotemporal role for ICP27 in regulating aberrant splicing. Through its interaction with the potent adaptor SRSF3, which is localized at nuclear speckles and binds to the exonic ICP27/SRSF3-responsive motif near the 5’ splice site of the targeted mRNA, ICP27 prevents spliceosome formation in a sequence-specific manner, thereby reducing splicing efficiency (Fig 6). The biological functions of the RGG RNA-binding domain, which binds to GC-rich RNAs, and of the NES domain, which interacts with NXF1, are partially overlapping in regulating pre-mRNA splicing. Mutation of either the RGG domain or the NES domain only partially impacted ICP27’s function (Fig 2 and 3). When the RGG RNA-binding domain is absent, ICP27 binds to targeted mRNAs indirectly via SRSF3 and promotes nuclear export of targeted mRNAs via interaction with NXF1 via its NES. When the NES domain is absent, ICP27 binds to targeted mRNAs directly and promotes nuclear export of targeted mRNAs interacts with NXF1 via the SRSF3 adaptor. However, dual mutation of the RGG domain and NES abolished the virus’s role in splicing inhibition of targeted mRNAs, expression of ICP27-dependent viral genes and viral growth (Figs 2 and 3), suggesting an essential role of ICP27 and SRSF3-mediated nuclear export of aberrantly processed mRNAs in ICP27-mediated splicing inhibition. This is further supported by ICP27’s counteraction of the U1 snRNP-mediated restrictive function of mRNA surveillance of aberrantly processed viral transcripts (Fig 5). These results suggest that ICP27-mediated counteraction of U1 snRNP contributes not only to splicing inhibition but also to RNA stability and expression of ICP27-dependent transcripts. It is also plausible that through interacting with SRSF3, ICP27 binds to intronic ICP27/SRSF3-responsive motifs upstream of the proximal intronic polyadenylation signal (PAS), thus blocking SRSF3’s role in inhibition of proximal PAS usage and also influencing interaction of ICP27 with CPSF, promoting intronic polyadenylation. Therefore, our results highlight an essential role of ICP27 together with the potent adaptor SRSF3 to counteract U1 snRNP’s role in pre-mRNA splicing, intronic polyadenylation, and nuclear export of aberrantly processed mRNAs, resulting in optimal viral gene expression during lytic infection. Our model also implies that during viral latency, when ICP27 (or the NES and RGG domain of ICP27) is absent, randomly activated antigenic viral transcripts are detained, spliced or destroyed, contributing to repression of randomly activated antigenic viral genes to achieve immune evasion.

## Discussion

Using splicing reporters and genetic analyses, we have identified key host factors and critical domains of HSV ICP27 that are required for ICP27-mediated gene/sequence-specific splicing inhibition, revealing a spatiotemporal mechanism for ICP27-mediated co-transcriptional pre-mRNA splicing. We showed that ICP27 hijacks SRSF3 to regulate sequence-specific alternative splicing and to promote nuclear export of unspliced mRNA targets in a sequence-specific manner via NXF1, which interacts with both SRSF3 and the N-terminal NES of ICP27. We have demonstrated that splicing inhibition through co-opting SRSF3 and subsequent nuclear export of unspliced mRNA mediated by ICP27 and SRSF3 are two separate steps essential for ICP27-mediated aberrant splicing and viral growth. Our results highlight that counteraction of U1 snRNP by ICP27 via co-opting SRSF3 plays a central role in ICP27-mediated aberrant splicing and intronic polyadenylation, as well as expression of ICP27-dependent viral genes.

### Interaction with SRSF3 enhances the target specificity and efficiency of ICP27-mediated aberrant splicing

Earlier studies suggested that ICP27 interacts with U1 snRNP and SAP145, a component the SF3b subcomplex of U2 snRNP, inhibiting splicing by preventing spliceosome assembly without target specificity [17,20,22,65,66]. It was also proposed that by binding to SRPK1, ICP27 redistributes the kinase from the cytoplasm into the nucleus, which causes SR proteins to become hypo-phosphorylated and thus inhibiting host global pre-mRNA splicing [17]. However, these models did not explain results observed in cells infected with WT and ICP27 mutant viruses. For example, in co-transcriptional splicing, ICP27 has no impact on splicing of β-globin pre-mRNA, a pre-mRNA substrate commonly used to demonstrate ICP27’s inhibitory role in pre-mRNA splicing in cell-free *in vitro* splicing experiments [9,18]. When expressed in cells, ICP27 appears to inhibit splicing in a gene/sequence-specific manner, affecting only a small percentage of cellular genes. More importantly, ICP27-mediated splicing inhibition is not dependent on its RGG RNA-binding domain, which binds to SRPK-1, suggesting that SRPK-1 is unlikely the critical factor and a unknown adaptor protein is involved in ICP27-mediated co-transcriptional aberrant splicing (Fig 1 and 2) [9,10,12,18].

Although ICP27 has been reported to interact with multiple serine/arginine rich (SR) proteins, only the interaction between ICP27 and SRSF3, initially identified through yeast-two-hybrid screening, has been confirmed as a direct interaction [17]. SRSF3 is one of the smallest SR proteins. Each SR protein contains a C-terminal arginine/serine domain that functions primarily in protein–protein interactions with other SR proteins or SR protein-related polypeptides such as U1-70K or U2AF35, and one or two N-terminal RNA-binding domains that bind to specific RNA sequences [67–69]. SR proteins bind to splicing enhancers located in exons and introns and promote the recruitment of U1 snRNP to 5’ splice sites and U2AF35 to 3’splice sites. Due to differences in the RNA binding domains among different SR proteins, each SR protein has different RNA binding specificity. Interestingly, the SRSF3-responsive motif identified using different methods closely resembles the previously studied ICP27-responsive motif, sharing a core cytosine-rich sequence [9,49–51]. Our results strongly support that among the SR proteins that may interact with ICP27, SRSF3 is the most potent adaptor used by ICP27 to promote sequence-specific splicing inhibition (Fig 1). These results also highlight that ICP27-mediated blocking of U1 snRNP binding to the 5’ splice site through direct or indirect binding to the ICP27/SRSF3-responsive motif near the 5’ splice site is a key element of ICP27-mediated aberrant mRNA splicing.

### Interaction with SRSF3 may also contribute to sequence-specific intronic polyadenylation

Reduced binding of U1 snRNP also relieves its inhibition of CPSF binding to the proximal PAS, thus promoting expression of short intronless viral and host transcripts prematurely cleaved and polyadenylated at the intronic PAS [9,10,60,70,71]. It has been proposed that ICP27 can directly bind to GC-rich sequences near the PAS, affecting its interaction of ICP27 with CPSF and thereby promoting polyadenylation of viral genes [9,13,72,73]. The ICP27-targeted host intronic polyadenylated transcripts also contain a C-rich motif that closely resembles the SRSF3 responsive motif [9]. Therefore, interaction of ICP27 with SRSF3, which binds to the intronic ICP27/SRSF3 responsive motifs upstream of the intronic PAS, may affect the interaction of ICP27 with CPSF, contributing to premature termination and cleavage of ICP27-targeted transcripts at the proximal intronic polyadenylation site. Moreover, it has been shown that the binding of SRSF3 upstream of proximal PASs inhibits the proximal PAS usage, leading to increased distal PAS usages [74]. Thus, interaction with ICP27 with SRSF3 also likely blocks SRSF3’s role in the inhibition of proximal PAS usage. Indeed, we have previously shown that the RGG RNA-binding domain mutant (d4-5), which cannot bind to the mRNAs directly but can interact indirectly via SRSF3, can still promote intronic polyadenylation of TMEM245, an ICP27-targeted cellular transcript that encodes a transmembrane protein, although to a significant lesser extent as compared with the WT HSV-1 [9]. It remains to be determined to what extent interaction with SRSF3 contributes to ICP27-mediated intronic polyadenylation.

### ICP27-mediated nuclear export of unspliced mRNA targets is required for its role in regulating co-transcriptional splicing, and SRSF3 can complement ICP27’s NES’s role in promoting nuclear export of aberrantly processed mRNAs

ICP27 was previously shown to promote nuclear export of intronless viral mRNAs via interaction with nuclear-export factor NXF1 and with splicing factors including SRSF3 and SRSF7 [19,31]. However, it was not known whether the nuclear export function of ICP27 is also required for its role in pre-mRNA splicing. We now demonstrate that ICP27-mediated aberrant splicing is not only dependent on its role in the prevention of spliceosome formation, but also dependent on its role in promoting nuclear export of aberrantly processed mRNAs. Mutation of both the NES (a critical domain required for binding to NXF1) and the RGG RNA binding domain abolished ICP27-mediated splicing inhibition in transfected or infected cells (Fig 2–4). Importantly, the dual mutant (d1-2/4–5) completely lost its role in the nuclear redistribution of NXF1, including its redistribution to the nuclear membrane (Fig 4B and D). Together with the impact of the NXF1 siRNA knockdown on the expression of ICP34.5β encoded by the unspliced full-length ICP34.5 mRNA (Fig 4E), these data strongly suggest that NXF1-mediated nuclear export plays a critical role in ICP27-mediated splicing inhibition. Indeed, all ICP27-targeted mRNAs contain suboptimal splice sites, and optimization of the splice site to canonical splice sites abolished ICP27-mediated splicing inhibition, suggesting a competition between spliceosome formation and ICP27’s counteraction [9]. If the unspliced pre-RNAs remain in nuclear speckles for an extended period due to inefficient transport when ICP27 (or its NES and RGG domain) is absent, this likely leads the unspliced pre-RNAs to be spliced or degraded by the nuclear mRNA surveillance machinery, contributing to the observed increased splicing efficiency in ICP27 mutants with mutations in the NES and RGG domain (Fig 2 and 3).

Viruses with mutations in either the NES (d1-2) or the RGG (d4-5) show a modest growth deficiency in Vero cells and a severe growth deficiency in HeLa cells, especially for the d1-2 mutant (Fig 3), which is generally consistent with a recent observation that reveals cell-type dependence in replication [56]. It appears that the function of NES sequences (via direct interaction with NXF1) and that of the RGG sequences (via direct interaction, with RNA targets) likely provide some redundancy in mediating ICP27 function, with the help of the potent adaptor SRSF3. As illustrated in Fig 6, our results suggest that SRSF3, through its interaction with the CTD of ICP27, can largely compensate for the requirement of the role of ICP27 NES, which interacts with NXF1, in nuclear export of ICP27-targeted mRNAs (Figs 2 and 3). Moreover, SRSF3, through directly binding to the SRSF3/ICP27 motifs, can partially compensate for the RNA-binding and SRPK-1-binding functions of the ICP27 RGG domain, facilitating nuclear export of these RNAs in a manner dependent on the NES and CTD of ICP27 (Figs 2 and 3). Thus, it appears that SRSF3 affects ICP27’s target specificity at both the pre-mRNA splicing level and nuclear export level (Figs 1, 2 and 4). Since SRSF3 shows variable expression across different cell types [42], the observed cell-type dependence of the NES and RGG domain for expression of ICP27-dependent genes may be due to differing expression levels of SRSF3. Nevertheless, dual mutations in both NES and RGG domains abolished ICP27-mediated splicing inhibition and promoted protein expression from ICP27-targeted unspliced transcripts to a level comparable with the inactive M15 mutation (Fig 2 and 3), strongly supporting that ICP27-mediated nuclear export of unspliced mRNA targets is required for its role in regulating co-transcriptional pre-mRNA splicing.

### U1 snRNP, which binds to the intact 5’ splice sites of aberrantly processed mRNAs, negatively regulates expression of ICP27-dependent viral genes

High-throughput splicing analysis revealed hundreds of novel alternative splice junctions and abundant cryptic 5’ and 3’ splice sites that map to previously unknown spliced genes in the absence of ICP27 [10]. Thus, ICP27-targeted viral transcripts and host intronic polyadenylated transcripts can be considered as aberrantly processed transcripts, which are normally subject to mRNA quality surveillance and nuclear exosome degradation. Facilitation of nuclear export of unspliced mRNA targets by ICP27 is an essential step for ICP27-mediated aberrant splicing, suggesting these aberrantly processed mRNA targets contain factors that would otherwise lead them to be detained in nuclear speckles and nucleus, and spliced or degraded in the absence of ICP27. Indeed, some ICP27-dependent transcripts, such as gC (whose splicing is also regulated by ICP27), are extremely unstable in the absence of ICP27 [14,15]. Two adaptors, the nuclear exosome-targeting (NEXT) complex and the poly(A) exosome-targeting (PAXT) connection, have been shown to facilitate recognition of specific targets. The NEXT pathway is involved in targeting the exosome to short, non-sequence-specific and unpolyadenylated RNA, while, the PAXT pathway is mainly involved in decay of polyadenylated RNA [62,75–77]. Recent studies suggest that the presence of intact 5’ splice sites, which are recognized by U1 snRNP, along with m6A modifications and polyadenylation factors, as nuclear RNA degradation codes in host intronic polyadenylated transcripts, lead to sequestration of these aberrantly processed transcripts in nuclear speckles, and subsequent degradation within the nucleus by ZFC3H1, a key component of the PAXT pathway [61,63,78,79]. All ICP27-mediated aberrantly processed targets contain intact 5’ss and many ICP27-targeted transcripts also contain m6A modifications [9,10,80]. It is likely that many ICP27-targeted viral genes and ICP27-activated host intronic polyadenylated transcripts are subject to PAXT surveillance when ICP27 is not present as all these transcripts contain intact 5’ splice sites [9,10,80]. Reduced expression of U1-70K promotes ICP27-dependent gene expression (Fig 5), further indicating that ICP27-dependent viral genes are likely subject to PAXT surveillance. The detailed mechanism by which ICP27 counteracts U1 snRNP remains to be further studied. It is plausible that ICP27 through interaction with SRSF3 blocks the binding of U1 snRNP to the impacted 5’ splice sites, in a way consistent its role in inhibition of the spliceosome formation. Overall, ICP27-mediated efficient nuclear export outpaces U1 snRNP-mediated degradation of aberrantly processed RNA that contains intact 5’ splice sites. In addition, SRSF3 was also shown to be functionally connected to the NEXT pathway for intronless mRNA decay [40]. Thus, SRSF3 may function as an adaptor that bridges ICP27-dependent targets in a sequence-specific manner to the nuclear exosomes for degradation when ICP27 is absent. Therefore, depending on the nature of restrictive factors encoded in these transcripts, different ICP27-dependent viral transcripts are likely regulated differently by the PAXT and NEXT pathways, contributing to cell type-dependent phenotypes as shown in Fig 3 and as recently reported [56]. This may also help to partially explain why the intronic polyadenylated transcripts mediated by ICP27 only partially overlap those activated by an inhibitor (antisense oligo) of U1 snRNP [11], since ICP27-mediated intronic polyadenylation is a combined outcome of its roles in spliceosome formation, polyadenylation, nuclear export and decay of these aberrantly processed mRNAs. Increased expression of ICP27 at both early and late infection in the U1-70K knockdown cells further suggests that U1-70K plays a role in the expression of ICP27 itself (Fig 5A, B and D). This observation is consistent with previous observations that ICP27 promotes expression of its own mRNA [13]. Moreover, in NXF1- or SRSF3-knockdown cells, ICP27 expression levels were increased (Fig 1F, 4E and F), implying a restrictive role of SRSF3 and NXF1 in the expression of ICP27 and ICP27-dependent genes. Further studies are needed to understand the role of SRSF3, NXF1 and U1-70K as well as other potential restrictive factors in determination of the fate of ICP27-dependent aberrantly processed transcripts in the absence and presence of ICP27.

Unlike many other viruses, the HSV-1 life cycle contains both a lytic-infection phase and a latent-infection phase. During the lytic phase, a coordinated temporal cascade of viral gene expression is initiated by viral immediate early genes including ICP27 to achieve efficient expression of viral genes. During the latent phase, expression of viral genes is repressed, and the only readily detectable viral transcripts are the latency-associated transcript (*LAT*) and LAT-encoded miRNAs [1–3]. Identification of SRSF3 as a potent adaptor for ICP27 not only provides a mechanism by which ICP27 inhibits splicing and promotes expression of viral target genes, ensuring the quality (correctness of the functional coding sequence) and quantity of viral genes during lytic infection, but also sheds light on the mechanism by which HSV takes advantage of host pre-mRNA processing machineries to restrict expression of randomly activated viral antigens to achieve optimal immune evasion during latency, when ICP27 is absent. Indeed, although ICP27 has clear homologs in all characterized mammalian herpesviruses, ICP27 functions very differently from its homologs in the regulation of pre-mRNA splicing. For example, in contrast to other ICP27 homologs, including those in VZV, HCMV and bovine herpesvirus 4, ICP27 has a unique role in promoting both mRNA accumulation and intron retention of the HSV gC mRNA [14]. ICP27’s homologs in human gamma herpesviruses, KSHV ORF57 and EBV SM (EB2), have also been reported to interact with SRSF3 [43,81]. However, their roles in regulating alternative splicing are very different from that of ICP27. For example, both ORF57 and EBV SM promote splicing of the KSHV K8β splicing reporter by attenuating the suppressive activity of SRSF3; in contrast, ICP27 is inactive in this assay [43,82]. Furthermore, EBV SM activates the alternative splice site of cellular STAT1 by recruiting and enhancing the activity of SRSF3 [81]. In contrast, SRSF3 does not appear to play a significant role in the basal splicing of ICP27-targeted genes (Fig 1), suggesting that unlike its homologs in gamma herpesviruses, ICP27-mediated splicing inhibition is not achieved by blocking or enhancing SRSF3’s activity. Instead, this interaction may help ICP27 specifically access the targeted intron, thereby improving the efficiency of its splicing inhibition. A notable structural difference is that ICP27 interacts with SRSF3 via its C-terminal domain, while KSHV ORF57 and EBV SM use their N-terminal domains [17,43,81]. The functional importance of ICP27’s C-terminal domain is underscored by the finding that its mutation abolishes splicing inhibition, whereas mutations in the RNA-binding domain only partially reduce inhibition efficiency without affecting target specificity (Fig 1 and 2). As a result, the interaction between ICP27 with SRSF3 appear to be relatively weak and transient since ICP27 does not relocate SRSF3 from nuclear speckles to the viral replication compartment, which contrasts with the interaction between ICP27 and ALY/REF or the interaction between EBV SM protein and SRSF3 [19,35,81]. Further investigation of the details of ICP27-mediated aberrant pre-mRNA processing will likely yield insight into both mechanisms of viral pathogenesis, potentially leading to identification of new targets for antiviral strategies and the design of novel viral vectors, as well as into the mechanisms by which the cell itself controls alternative polyadenylation and splicing of selected genes.

## Methods

### Cells, virus and antibodies

293T, HeLa and Vero cells were obtained from ATCC. Sh-U1-70K shRNA and NS-shRNA knockdown stable cells were prepared by infection of HeLa cells with lentiviruses encoding U1-70-specific shRNA (TTCGTGGCGAGAGTGAATTAT) or non-specific scramble shRNA#1 (CCTAAGGTTAAGTCGCCCTCG) (VectorBuilder). Cells were selected for 2 weeks under puromycin treatment. HSV-1 strain KOS, HSV-1 *ICP27* mutant viruses, including d1-2, d4-5, m15 and d27-1, and the V27 *ICP27*-complementing Vero cell line used to grow *ICP27* mutant viruses were obtained from Dr. Stephen Rice (University of Minnesota) [53,54,83]. d1-2/4–5, containing deletions in both d1-2 and d4-5, and d4-5/LeuM, containing the same deletion in d4-5 and two point-mutations of leucine 11 and 13 to alanine (Fig 2), were constructed by VectorBuilder using KOS bacterial artificial chromosome (BAC) system. The mutations were confirmed by sequencing. The BAC backbone in both the WT and mutations were removed by recombination to prior to be amplified in V27 cells. Anti-HSV ICP4, ICP8, gD, U1-70K, b-tubulin antibodies (Santa Cruz), anti-HSV ICP27 (H1113), anti-gC (Abcam), anti-NXF1, b-actin (Cell Signaling), SRSF3 (Proteintech), anti-cMyc monoclonal antibody (Sigma) and anti-Flag M2 antibody (Sigma) were sourced commercially. Anti-HSV-1 ICP34.5 antibody was obtained from Dr. Bin He [84]. Anti-HSV-2 ICP34.5 was described previously [2]. Vero and HeLa cells were infected in biological duplicate at an MOI of 5 and incubated for 24 hours. The amount of viral progeny in each culture was determined by plaque assay of the harvested cell lysates on ICP27-complementing V27 cells [53,85].

### Plasmids, primers and siRNAs

pHSV-2 ICP34.5, encoding the 5’ UTR and entire HSV-2 ICP34.5 coding sequences, and pICP27, an HSV-2 expression vector, was described previously [18]. pFlag-ICP27, pFlag-d1-2 (p-d1-2), pFlag-d4-5 (p-d4-5), and pFlag-M15 (p-M15), encoding wild-type (WT) and mutant HSV-1 ICP27 sequences with an in-frame Flag tag epitope in Vector E1775 (Sigma) were subcloned by PCR amplification with corresponding primers (S1 Table) and enzymatic digestion from corresponding HSV-1 ICP27 WT and mutant plasmids provided by Dr. Stephen Rice [53,83]. Flag-tagged HSV-1 ICP27 mutant plasmids including p-SerM, p-d1-2/4–5, p-d4-5/SerM, p-dLeu/4–5, p-dAC/4–5, p-dAC2/4–5, and p-d4-5/LeuM were generated by overlapping PCR with specific primers (S1 Table) using the corresponding WT or ICP27 single mutant plasmids (described above) as templates. Primers and synthesized DNA fragments used to construct pK8 splicing reporters and ICP27 mutants are described in S1 Table. HSV-2 *ICP27* mutant plasmids, including p∆R2 and pM15, were obtained from Dr. Masatoshi Hagiwara (Tokyo Medical and Dental University) [12]. pGFP-SRSF3 (Genscript) and pMyc-NXF1 (GeneCopeia) were sourced commercially. Nonspecific siRNA (NS-siRNA) and siRNA smart pools specific to SRSF3, SRSF7, and NXF1 were obtained from Dharmacon. 293T cells were transfected with 20 nM siRNA. At 48 hours post transfection, cells were transfected with plasmids or infected with viruses indicated in the figure.

### Western blot, RT-PCR and Northern blot analysis

293 cells, HeLa cells or Vero cells were infected with viruses indicated in the corresponding figures at a multiplicity of infection (MOI) of 5. Total protein or RNA were prepared at different time points post inoculation. Western blot was performed using the antibodies described above. For RT-PCR, total RNAs were extracted using All-Prep DNA/RNA kits (Qiagen). The primer sequences are listed in S1 Table. 293 cells were transfected with plasmids using Lipofectamine 2000 (Thermo Fisher). Total protein or RNAs were prepared 24 hours post transfection.

### Immunofluorescence staining of HSV-1-infected HeLa cells

HeLa cells grown on eight-well chamber slides (Fisher Scientific, MA) were transfected with or without pGFP-SRSF3 and/or pMyc-NXF1 as indicated in the figure using Lipofectamine 2000 as per the manufacturer’s guidelines. At 40 hours post-transfection, cells were infected with or without WT HSV-1 (KOS strain), d4-5, d4-5/LeuM, d1-2/4–5, or m15 at a multiplicity of infection (MOI) of 5. At 6 h postinfection (hpi), cells were fixed with 4% paraformaldehyde, permeabilized with PBS containing 0.1% Triton X100, and blocked with 10% goat serum in PBS containing 0.1% Triton X100. The cells wre incubated with the anti-ICP27 (H113) or the anti-Myc tag monoclonal antibody overnight, followed by washing and incubation with the Alexa Fluor 594-conjugated goat anti-mouse IgG. The stained cells were washed, and nuclei were stained using Hoechst 33342 (ThermoFisher Scientific). The chamber slides were examined using Leica TCS_SP8 DMI6000 confocal microscope system (Leica Microsystems, Mannheim, Germany) with a 100x oil objective at the CBER Core facility. Fluorescence colocalization and line measurement analyses were performed using the Leica LASX image analysis software. Pearson’s co-localization coefficient (PCC) analyses were performed using the Leica LASAF and Bitplane Imaris software. Briefly, mean fluorescence intensity was measured by averaging readings from 4-8 cells with positive signals for Figs 4A and 8–15 images with positive signals for 4B per treatment group. The results are presented as means ± SD. The data were analyzed by one-way ANOVA at the 95% confidence interval using Prism (version 10) software for Windows (GraphPad Software, San Diego, CA).

## Supporting information

S1 Table Sequences of oligonucleotide primers, probes and point mutations for splicing reporter genes. (DOCX)
