# Supplementary material for: HSV ICP27 hijacks host splicing factor SRSF3 to regulate pre-mRNA splicing and export for viral gene expression and immune evasion
Source: PLoS Pathog. 2026 Apr 10;22(4):e1014146. doi: 10.1371/journal.ppat.1014146 (PMC13089877; doi:10.1371/journal.ppat.1014146)
Supplement: S1 Table — (DOCX) [file ppat.1014146.s001.docx]

**Table S1. Sequences of oligonucleotide primers, probes and point mutations for splicing reporter genes.**

| **Name of Genes** | **Name of Primers** | **Sequence of the Primers** |
| --- | --- | --- |
| K8 reporter  forward | oST1011 | AAGCTTGAATATGTGATCAGTCACATTCT |
| K8 reporter  reverse | oST1012 | CAGCATGTCGCGAAGGAAAATAATC |
| HSV-2 ICP34.5  forward | oST432 | GAGCCCAGCCGCCCGCCATGT |
| HSV-2 ICP34.5  reverse | oST426 | GGTTCAACCCTAGACCGCCCGACGG |
| HSV-1 IC27 (for mutant plasmid)  forward | oST1294 | CCATGTGTAGCCTGGATCCCAACGA |
| HSV-1 ICP27 (for reverse plasmid)  reverse | oST1297 | GCCATGAGCAAAGAAGGACAACACGTG |
| d1-2 forward mutant primer | oST1298 | TGCTAATTGACCTCCTCGACGCCGCTCGCCCGGCGGT |
| d1-2 reverse mutant primer | oST1199 | GGCGAGCGGCGTCGAGGAGGTCAATTAGCATATCAATGT |
| LeuM forward mutant primer | oST1309 | TTGATATGCTAATTGACGCTGGCGCTGACGCACCGACAGCGATCTGGACGA |
| LeuM reverse mutant primer | oST1310 | TTGATATGCTAATTGACGCTGGCGCTGACGCACCGACAGCGATCTGGACGA |
| LeuM/d4-5 (forward primer for pFlag vector) | oST1103D | GAATTCCATGGCGACTGACATTGATATGGCAAT |
| ICP27 mutant  Reverse primer for plag vector) | oST1104 | TGTACCTAAAACAGGGAGTTGCAATAA |
| ICP27 forward primer for pFlag vector | oST1103 | GAATTCCATGGCGACTGACATTGATATGCTAATTG |
| dLeu/4-5 forward mutant primer | oST1308 | ATGGCGACTGACATTCTGGACGAGGACCCccccga |
| dLeu/4-5 reverse mutant primer | oST1307 | GGGTCCTCGTCCAGAATGTCAGTCGCCAT |
| dLeu/d4-5 (forward primer for pFlag vector) | oST1103C | GAATTCCATGGCGACTGACATTCTGGACGA |
| dAC/4-5 forward mutant primer | oST1311 | GGCGAGCGGCGTCGAGCAGATCGCTGTCGGAGAGGT |
| dAC/4-5 reverse mutant primer | oST1312 | ACCTCTCCGACAGCGATCTGCTCGACGCCGCTCGCCCGGCGGT |
| dAc2/4-5 forward mutant primer | oST1316 | CGACAGCGATCTGCACGGAGAGGACGGACCGGA |
| dAc2/4-5 reverse mutant primer | oST1315 | CGTCCTCTCCGTGCAGATCGCTGTCGGAGAGGT |
| SerM/4-5 forward mutant primer | oST1296 | GCCGACGCAGATCTGGACGAGGACCCCCCCGAGC |
| SerM reverse mutant primer | oST1295 | TCCAGATCTGCGTCGGCGAGGTCCAGGCCGAGGT |
| UL5 (spanning intron)  forward | oST1025 | GCAACCGCACGCTGCGCGAGTA |
| UL5 (spanning intron)  reverse | oST1026 | CGAGGGTCCTCTGGGTCGCATCCA |
| UL4 forward | oST1029 | CACCGCGAGATGTCCAATCCACAG |
| UL4 reverse | oST1030 | GCGAGGAGCTGACCCAGGAGTCGA |
| Beta-actin (exon 4)  forward | oST728 | GACCTGTACGCCAACACAG |
| Beta-actin (exon 5)  reverse | oST719 | TCGTCATACTCCTGCTTGC |
| GAPDH (exon 2)  forward | oST726 | TACATGTTCCAATATGATTC |
| GAPDH (exon 3)  reverse | oST727 | GTGGACTCCACGACGTACTC |
| BCL-XL  forward | oST724 | CATGGCAGCAGTAAAGCAAG |
| BCL-XLs  Reverse | oST725 | GCATTGTTCCCATAGAGTTCC |
| *ATXN2L* exon 19 forward | oST921 | CCAGGCCATCGTGTCATCCTCTAC |
| *ATXN2L* exon 20 reverse | oST922 | TGCGGCTGGCTTCCAGTAGG |
| ATNX2L exon 16 forward | oST949 | CCATCCCGGTGCTGACAGCAG |
| ATNX2L exon 17 reverse | oST950 | CCAGGCACTGAATTGGATACAGGAT |
| Synthesized DNA for pK8-SRSF3 reporter(HindIII/EcoRI) |  | AttaagcttGaagtatgtgatcagtcacattctcccacgcgaaagcaaggcagatacggCCTCGTCCCCTCGTCCCCTCGTCCcagcaggtatagacgggaaacaggtgtctatcttggccggctggttactcaaatgggaacaatggcgccaccttgctgtctttgtaggcattagaagaaaaggatgcacaactatgtttcctagcggcgagattggaggcacataaggaacagattattttccttcgcgacatgctgGAATTCtaa |
| Synthesized DNA for pK8-2xSE4 reporter(HindIII/EcoRI) |  | AttaagcttGaagtatgtgatcagtcacattctcccacgcgaaagcaaggcagatacggCTGCACCACCACCTATCTATTCTGCACCACCACCTATCTATTcagcaggtatagacgggaaacaggtgtctatcttggccggctggttactcaaatgggaacaatggcgccaccttgctgtctttgtaggcattagaagaaaaggatgcacaactatgtttcctagcggcgagattggaggcacataaggaacagattattttccttcgcgacatgctgGAATTCtaa |
| Synthesized DNA for pK8-SRSF1 reporter(HindIII/EcoRI |  | AttaagcttGaagtatgtgatcagtcacattctcccacgcgaaagcaaggcagatacggAGAAGAACTCTGAGGAATCAGAGGAAGAAGAAcagcaggtatagacgggaaacaggtgtctatcttggccggctggttactcaaatgggaacaatggcgccaccttgctgtctttgtaggcattagaagaaaaggatgcacaactatgtttcctagcggcgagattggaggcacataaggaacagattattttccttcgcgacatgctgGAATTCtaa |
| Synthesized DNA for pK8-SRSF7 reporter(HindIII/EcoRI) |  | AttaagcttGaagtatgtgatcagtcacattctcccacgcgaaagcaaggcagatacggAGACGACGACctAgacAGACGACGACctAgacagcaggtatagacgggaaacaggtgtctatcttggccggctggttactcaaatgggaacaatggcgccaccttgctgtctttgtaggcattagaagaaaaggatgcacaactatgtttcctagcggcgagattggaggcacataaggaacagattattttccttcgcgacatgctgGAATTCtaa |
| Synthesized DNA for pK8-SRSF5 reporter(HindIII/EcoRI) |  | AttaagcttGaagtatgtgatcagtcacattctcccacgcgaaagcaaggcagatacggTGGGAGCagtcGGCTCGTTGGGAGCagtcGGCTCGTcagcaggtatagacgggaaacaggtgtctatcttggccggctggttactcaaatgggaacaatggcgccaccttgctgtctttgtaggcattagaagaaaaggatgcacaactatgtttcctagcggcgagattggaggcacataaggaacagattattttccttcgcgacatgctgGAATTCtaa |
| Synthesized DNA for pK8-SRSF2 reporter(HindIII/EcoRI) |  | AttaagcttGaagtatgtgatcagtcacattctcccacgcgaaagcaaggcagatacggGGCCCCTGGGCCCCTGGGCCCCTGcagcaggtatagacgggaaacaggtgtctatcttggccggctggttactcaaatgggaacaatggcgccaccttgctgtctttgtaggcattagaagaaaaggatgcacaactatgtttcctagcggcgagattggaggcacataaggaacagattattttccttcgcgacatgctgGAATTCtaa |
|  |  | AttaagcttGaagtatgtgatcagtcacattctcccacgcgaaagcaaggcagatacggCTGCACCACCACCTATCTATTCTGtgtCACtgtCTATCTATTcagcaggtatagacgggaaacaggtgtctatcttggccggctggttactcaaatgggaacaatggcgccaccttgctgtctttgtaggcattagaagaaaaggatgcacaactatgtttcctagcggcgagattggaggcacataaggaacagattattttccttcgcgacatgctgGAATTCtaa |
| Synthesized DNA for pK8-SE4M1 reporter(HindIII/EcoRI) |  | AttaagcttGaagtatgtgatcagtcacattctcccacgcgaaagcaaggcagatacggCTGCACCACCACCTATCTATTCTGtgtCACtgtCTATCTATTcagcaggtatagacgggaaacaggtgtctatcttggccggctggttactcaaatgggaacaatggcgccaccttgctgtctttgtaggcattagaagaaaaggatgcacaactatgtttcctagcggcgagattggaggcacataaggaacagattattttccttcgcgacatgctgGAATTCtaa |
| Synthesized DNA for pK8-SE4M2 reporter(HindIII/EcoRI) |  | AttaagcttGaagtatgtgatcagtcacattctcccacgcgaaagcaaggcagatacggCTGtgtCACtgtCTATCTATTCTGtgtCACtgtCTATCTATTcagcaggtatagacgggaaacaggtgtctatcttggccggctggttactcaaatgggaacaatggcgccaccttgctgtctttgtaggcattagaagaaaaggatgcacaactatgtttcctagcggcgagattggaggcacataaggaacagattattttccttcgcgacatgctgGAATTCtaa |
| Synthesized DNA for pK8-CCT reporter(HindIII/EcoRI) |  | Attaagctt/Gaagtatgtgatcagtcacattctcccacgcgaaagcaaggcagatacggcc**C**c**C**tgtc**C**tcccca**C**c**C**t**C**c**C**caagac**CC**ct**C**c**C**gcag/gtatagacgggaaacaggtgtctatcttggccggctggttactcaaatgggaacaatggcgccaccttgctgtctttgtag/gcattagaagaaaaggatgcacaactatgtttcctagcggcgagattggaggcacataaggaacagattattttccttcgcgacatgctg/GAATTCtaa |
